# Space for power: feeling powerful over others’ behavior affects peri-personal space representation

**DOI:** 10.1007/s00221-023-06719-1

**Published:** 2023-10-21

**Authors:** Tommaso Bertoni, Maria Paola Paladino, Elisa Pellencin, Silvia Serino, Andrea Serino

**Affiliations:** 1 MySpace Lab, Department of Clinical Neurosciences, University Hospital of Lausanne, University of Lausanne, Lausanne, Switzerland; 2 Department of Psychology and Cognitive Science, University of Trento, Rovereto, Italy; 3 Neurology V and Neuropathology Unit, Fondazione IRCCS Istituto Neurologico Carlo Besta, 20133 Milan, Italy; 4 Department of Psychology, Università Degli Studi Milano-Bicocca, Piazza Dell’Ateneo Nuovo, 1, 20126 Milan, MI Italy

**Keywords:** Power, Peri-personal space, Virtual reality

## Abstract

We investigated whether and how social power affects the representation of peri-personal space (PPS). We applied a multisensory interaction task to assess PPS representation and the Personal Sense of Power Scale to assess participants’ feelings of power over others’ behaviors and over others’ opinions. In Study 1, we probed PPS representation in a virtual social context. Participants with a higher sense of power showed a less defined differentiation between the close and far space as compared to participants with a lower sense of power. This effect was replicated in Study 2 when participants performed the task in a non-social context (with no person in the scene), but only after they were reminded of an episode of power. Thus, social power—the perception of power over others’ behavior—affects the multisensory representation of the self in space by blurring the differentiation between one’s own PPS and the space of others.

**Supplementary Information:**

The online version contains supplementary material available at 10.1007/s00221-023-06719-1.

## Introduction

Thirty feet is the distance that is automatically set around important public figures (Hall 1966, p.124).

This quote—taken from Hall’s seminal work on how people use space—suggests that people tend to keep further distance from powerful and high-status people. This behavior is generally interpreted as a sign of respect toward individuals in positions of power and authority, and as an indication that power affects self-representation in space. Powerful individuals are believed to “occupy more space” as if feeling powerful would magnify the spatial extension of the self in the area immediately surrounding the body—i.e., the peri-personal space. In the present research, we rely on the concept of peri-personal space (PPS) developed in neuroscience to empirically test the validity of this idea.

### Peri-personal space representation

It is not just anecdotal evidence, but also a scientific fact, that people treat the area immediately surrounding the body—i.e., the peri-personal space—as if it were a spatial extension of the self (Blanke et al. 2015; Noel et al. 2015b; Serino 2019). A consistent body of neurophysiology and neuroimaging studies has indicated that PPS is a multisensory representation of the space near the body—encoded by a set of neurons in the fronto-parietal areas—where tactile information on one’s body is preferentially integrated with visual or auditory information from external stimuli as they approach the body (Graziano and Cooke 2006; Cléry et al. 2015; Di Pellegrino and Làdavas 2015). At a behavioral level, this is clearly shown by performance in cross-modal interaction tasks in which participants are asked to react as soon as possible to a tactile stimulation delivered on one’s own body while receiving an external concurrent (auditory or visual) stimulus. Although irrelevant to the task, a stimulus presented at closer distances (i.e., within the peri-personal space) facilitates the processing of the tactile input compared to the same stimuli presented farther in space (Macaluso and Maravita 2010; Canzoneri et al. 2012; Serino et al. 2015a). Importantly, the extent of space coded by the brain as PPS is not fixed. The size and shape of PPS representation vary as a function of interaction with external objects or other people. For instance, PPS representation extends when reaching an object (Brozzoli et al. 2010), when walking forward (Noel et al. 2015a), or after using a tool to reach the far space (Canzoneri et al. 2013). When facing another person, to simulate the space of social interaction, PPS representation adjusts to the type of relationship. When the partner of the relation is cooperative (Heed et al. 2010; Teneggi et al. 2013; Hobeika et al. 2019) or moral (vs. immoral) (Iachini et al. 2015a; Pellencin et al. 2018), PPS representation is more extended toward them. These findings suggest that PPS representation rapidly adjusts to the social context, too.

An intriguing and theoretically relevant question is at the core of the present research: whether PPS representation maps also onto some individual psychological dispositions and states. We investigated this question by examining PPS representation both in terms of its spatial extent (i.e., the PPS boundary) and its shape (i.e., differentiation between peri- and the extra-personal space resulting from the amount of multisensory processing allocated in the near vs. far space).

Concurrent available evidence supports this hypothesis. People, depending on individual differences, approach the world differently and this could also influence PPS representation, as this is ultimately a sensory–motor interface mediating individual–environment interaction (Serino 2019). Literature suggests that spatial representations largely varies across people, depending on different clinically relevant traits, such as schizotypal traits or anxiety (Sambo and Iannetti 2013; Iachini et al. 2015b; Di Cosmo et al. 2018; Ferroni et al. 2020). For instance, anxiety (measured by the State–Trait Anxiety Inventory, STAI; Spielberger et al. 1983) is linked to a larger individuals’ defensive PPS (Sambo and Iannetti 2013; Spaccasassi and Maravita 2020) or smaller reaching distance (Iachini et al. 2015b). The link between PPS extent and anxiety is in keeping with one of the key putative functions of PPS representation, namely, to anticipate potential contacts with threatening stimuli and to prepare defensive responses (Graziano and Cooke 2006). In the same perspective, Gherri and colleagues (Gherri et al. 2022) observed an inverse relationship between levels of empathy and the strength of multisensory integration within PPS, together with a reduced differentiation between peri- and extra-personal space. These results might indicate that individuals with higher levels of empathy tend to process self-related stimuli over a larger space in the presence of others.

Other findings in the same line of research suggest that psychological dispositions can also account for when and why PPS representation dynamically adjusts to the context. For instance, only when a threatening stimulus is present (e.g., dog, spider), PPS representation extends (Taffou and Viaud-Delmon 2014; Hobeika et al. 2019) and becomes more differentiated—in terms of multisensory processing—from the representation of the far space (de Haan et al. 2016). Importantly, these effects occur only in participants who are afraid of that specific threat. More recently, we have shown that the introduction of COVID-19-related social distancing measures resulted in a reduction of PPS extent and in a stronger near–far differentiation (Serino et al. 2021). Interestingly, individuals who were more afraid of being contaminated by pathogens (as evaluated with the Germ aversion subscale of the Perceived Vulnerability Scale (Duncan et al. 2009) and were more likely to take precautionary measures to avoid contacts had a stronger near–far differentiation.

As suggested above, research on individual psychological differences offers a unique perspective to investigate between-individuals variability in PPS representation, but also an opportunity to understand “when” and “why” PPS dynamically shapes to adapt to specific contexts. In the present research, we further investigated the role of individual psychological difference and examined in particular the influence of personal sense of power in PPS representation in both social (i.e., when facing another person) and non-social (i.e., in an empty corridor) context. In the further sections of the paper, we will first introduce the notion of the personal sense of power, relying mainly on research in social psychology, and then introduce possible hypotheses on how, when, and why feeling powerful would affect individuals’ PPS representation.

### Power as a psychological state

Power is a ubiquitous characteristic of the social and relational world. Power can be defined as the potential to influence others in psychologically meaningful ways (French et al. 1959; Guinote 2017). It is therefore a relational feature that is usually defined by the situation (e.g., working organization, family, etc.) and the roles people have in it (e.g., manager, subordinate, parent, child). Power can also be thought of as a psychological state, temporarily elicited by the context (e.g., the role), or stemming from the personality and therefore as a stable characteristic of the individual. In this regard, Anderson, John, and Kelter (Anderson et al. 2012) introduced the concept of a personal sense of power as the perception of one’s ability to influence others. Using a self-reported scale, they showed that some people simply feel more powerful than others, across situations. In the present research, we rely on this conceptualization to investigate the relationship between individual differences in the personal sense of power and the multisensory representation of PPS.

Whether it is elicited by roles or a stable self-perception, feeling powerful or powerless has a series of important downstream consequences on the individual’s affect, higher cognition, and motor behavior. For instance, powerful people have a more positive view of themselves (Wojciszke and Struzynska–Kujalowicz 2007) and a more optimistic perspective on events even if risky (Anderson and Galinsky 2006). Being powerless impairs executive functions (Smith et al. 2008), whereas being powerful increases the ability to ignore task-irrelevant information. Importantly for the present research, a high sense of power increases the tendency to take any action, whether this is in the service of personal desires or a pro-social act (Galinsky et al. 2003). For instance, participants primed with power are more likely to stop an annoying fan (Galinsky et al. 2003), but also more likely to run and help a victim in case of emergency (Baumeister et al. 1988) and to initiate an approach to motor response (Maner et al. 2010). Guinote (2017; Guinote and Chen 2018) has recently suggested a theoretical framework to integrate these findings into the literature. She suggests that power energizes the self and increases the goal-related approach motivation. Differently stated, powerful individuals are focused on their goal/s and act in a way that prioritizes the attainment of this goal. Thus, the effects of power on affect, cognition, and motor actions should be seen in relation to goal pursuit.

### The present research: feeling powerful affects PPS multisensory representation

In the present research, we relied on the concept of power as a psychological disposition to investigate whether individuals feeling powerful and powerless differ in their multisensory representation of PPS. Despite its intuitiveness, this idea, to the best of our knowledge, has never been empirically investigated. Feeling powerful is a noteworthy psychological individual characteristic to examine in the context of PPS representation for at least three reasons.

First, it would provide a better understanding of the role of individual dispositions in PPS representation. Research up to now has mainly investigated traits with clinical relevance (e.g., anxiety, schizophrenia and phobia), while studies that examine the role of non-clinical individual characteristics are needed. Second, it contributes to the ongoing debate on the nature and functions of the PPS representation. Studies have shown that multisensory representation of PPS is immediately translated into potential motor responses (Cooke and Graziano 2004; Makin et al. 2009; Serino et al. 2009; Finisguerra et al. 2015), either to protect the body from threat or to support appetitive actions (Rizzolatti et al. 1997; Ladavas and Serino 2008; Cléry et al. 2015; de Vignemont and Iannetti 2015; Serino 2019). Given that power is linked to the tendency to act and more generally to an approach motivation, the present research would provide indications concerning the action-related function of PPS representation.

Finally, the present research would extend our knowledge about the role of top-down and social processes in PPS representation and ultimately its functions in social interactions. While the research on PPS plasticity in human–object interaction and in response to sensory–motor inputs has a long tradition, only more recently the field has investigated the mutual relationship between PPS representation and social interactions. In this context, up to now, research has focused on morality (Iachini et al. 2015a; Pellencin et al. 2018), cooperation (Heed et al. 2010; Hobeika et al. 2019; Dell’Anna et al. 2021), and fairness (Teneggi et al. 2013), but never, to the best of our knowledge, on power. This is surprising as power is an omnipresent feature of our social world and, most importantly, a dimension that people spontaneously use to structure social relations (Fiske et al. 1991).

In the present research we conducted two studies in which we relied on a visual–tactile interaction task to assess individuals’ PPS representation (Canzoneri et al. 2012; Teneggi et al. 2013; Pellencin et al. 2018) and on the scale of the personal sense of power (Anderson et al. 2012) to identify powerful and powerless participants. Based on a recent validation work (Paladino et al. 2022), this scale allowed to capture two facets or domains of the personal sense of power and to differentiate participants, accordingly, on the self-evaluation of one’s own ability to have (1) power over others’ opinion and (2) power over others’ behavior. Study 1 assessed the representation of multisensory PPS in the context of a potential social interaction by means of mixed reality (Serino et al. 2018). Participants were asked to respond as soon as possible to a tactile stimulation on their body, while a virtual object was approaching at six different distances between the participants and another person facing them. Despite being instructed to focus on the tactile stimulation and to ignore the visual stimuli, several studies in the literature (for a comprehensive review, see Serino 2019) demonstrated that reaction times to touch increase as a function of the perceived distance of the external object at the time of tactile inputs. The distance between the visual stimulus and the participants' bodies at which this multisensory effect occurs is used as a proxy for PPS representation. This enables measuring the spatial extent of PPS representation as well as the degree of differentiation between near and far space. These PPS indexes were studied in relationship between individual differences in personal sense of power as assessed by the scale of the personal sense of power (Anderson et al. 2012). Study 2 was designed to investigate whether the effect of personal sense of power on PPS representation depends on contextual demands that make this individual disposition cognitively relevant and accessible. In Study 2, we did not probe the PPS representation in a social context—as the presence of another person would inevitably make this relational feature relevant. Instead, we assessed it in a non-social context (i.e., an empty corridor) before and after a manipulation aimed at priming their perceived power (i.e., asking to remind an episode related to high vs. low sense of power). As in Study 1, participants’ personal sense of power over others’ behavior and others’ opinions was also measured. This experimental design allows to test the role of cognitive accessibility of personal sense of power and at the same time provides indications on whether power affects PPS tout court, that is, independent of contextual demands.

## Study 1

### Materials and methods

#### Participants and inclusion criteria

We analyzed the data of 84 right-handed students (67 females; ages ranging from 18 to 33, *M* = 20.49 and SD = 1.90) at the University of Trento, Italy, who (1) participated in a larger project on PPS representation between November 2016 and April 2017 in our laboratory and (2) completed questionnaire measures and tasks relevant for the study (see the procedure) as a first task, that is, before receiving any other eventual experimental manipulation (for more information see Supplementary Materials, 1.1 Recruitment and data collection). Course credits were offered to volunteers and informed consent was obtained from all of them.

## Materials

The PPS task was administered with the aid of a virtual reality headset (Oculus Rift DK2; 900 × 1080 per eye, ~  105° FOV) and the RealiSM software (Reality Substitution Machine, http://lnco.epfl.ch/realism), a new augmented-reality technology developed at the Laboratory of Cognitive Neuroscience at the École Polytechnique Fédérale de Lausanne. This technology allowed us to integrate a pre-recorded real scene (a photo of a person in a corridor, see below) with a virtual element, a cube, and to administer the PPS task. The scales were presented, and responses were registered with the aid of online survey software.

## Procedure

Participants were invited to participate in a study on “the role of social and cognitive factors in social interactions”. The study consisted of two sessions: an online questionnaire, followed (from 7 to 40 days later) by a laboratory session. In the online questionnaire, participants responded to a series of questions and some scales including the Personal Sense of Power Scale and the Core Self-Evaluations Scale (generally used to assess self-esteem). Given that sense of power is generally related to self-esteem (Wojciszke and Struzynska–Kujalowicz 2007), in some additional analyses, we also included the score on this last scale to better establish the unique role of social power. In the laboratory session, participants performed a social PPS task in virtual reality (VR) and then responded to a questionnaire concerning the experience and their impression of the person they saw in the VR task. For some participants, the laboratory session continued and additional tasks were performed (see Supplementary Material). The scales and the tasks that were the focus of the present research are herein described in detail.

## Personal sense of power scale

The Italian version of the Personal Sense of Power Scale (Anderson et al. 2012; Paladino et al. 2022) was administered to the participants. Responses were registered on a seven-point scale (1 = totally disagree; 7 = totally agree). Based on a recent work (Paladino et al. 2022),^1^ we calculated the following two indices of the personal sense of power for each participant: the perception of one’s own (1) power over others’ opinion (2) and power over others’ behavior (see Supplementary Materials, 1.2 Personal sense of power).

## Core self-evaluations scale

The 12 items of the scale were administered to participants and responses were registered on a seven-point scale (1 = totally disagree; 7 = totally agree). This scale was developed by Judge and colleagues (Judge et al. 2003) and provides an appraisal of one’s worthiness, effectiveness, and capability as a person.

## The PPS social task

The task was identical to the visuo-tactile social PPS task used by Pellencin and colleagues (Pellencin et al. 2018). Participants sat at a desk, wore a head-mounted display, and held in their non-dominant hand a vibro-tactile stimulator (that administered the tactile stimuli) and in their dominant hand a computer mouse (so as to register the response to the tactile stimuli). Participants were instructed to respond to the tactile stimulation—as fast as possible—by pressing the button of the mouse and to ignore visual stimuli presented on the head-mounted display.

**Fig. 1:**
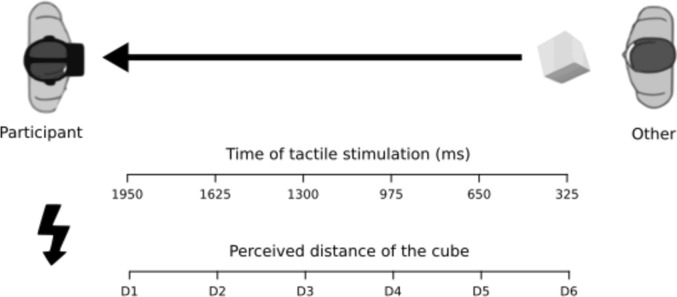
Study 1, experimental setup of the PPS task. Participants wore a head-mounted display through which they saw a pre-recorded movie of an actress standing in front of them in an empty corridor, at a distance of approximately 1.5 m. During each visuo-tactile trial, they received tactile stimulation in their left hand, while a task-irrelevant object (i.e., a virtual cube) loomed toward their face. The tactile stimuli were delivered in randomized order at different temporal delays (range: 325–1950 ms) from the beginning of the object movement and were thus perceived when the cube was at six different distances from the participants' faces (respectively, D6–D1). Participants were asked to ignore the visual stimulus and to respond to the tactile stimulation by pressing the mouse button. In unimodal (tactile only) trials, participants had to respond to tactile stimulation delivered at the same temporal delays, while no virtual cube was displayed. In catch trials, the looming cube was displayed, and no tactile stimulation was delivered

The task included three types of trials: bimodal visual–tactile, unimodal tactile and catch trials. The critical bimodal visuo-tactile trials started with a white fixation cross in the center of a black screen that disappeared after 300 ms. Then, participants saw a white corridor, where an actress (27 years old, neutral expression, wearing a white T-shirt and jeans) stood at a far location, at a distance of approximately 1.5 m. After 700 ms from the appearance of the actress, a tridimensional brown virtual cube (0.2 m × 0.2 m × 0.2 m) appeared, at the level of her neck. Then the cube started to move approaching the participant with a speed of 0.75 m/s, it moved for 2600 ms and then remained still for 400 ms at the end of its trajectory. The face of the actress was always visible. Together with the visual stimulus, a tactile stimulation—clearly above the perceptual threshold and lasting 350 ms—was delivered via a single vibro-tactile device that the participant held in his/her left hand for the duration of the task. Importantly, the tactile stimulation was given at six different temporal delays from the appearance of the cube (after 325, 650, 975, 1300, 1625, and 1950 ms) and thus perceived by the participant when the virtual object was placed at six distances from her/him (D1–D6) (Fig. 1). Specifically, when the vibro-tactile stimulation was delivered after 325 ms from the beginning of the movement of the cube, the cube was perceived at the farthest distance from the participant (D6). Conversely, when the vibro-tactile stimulation was given after 1950 ms, the cube was at the closest distance (D1). Differently stated, a longer delay corresponded to a closer object distance. In catch trials, the moving virtual cube and the other person in the corridor were shown, but no tactile stimulation was administered. In the unimodal tactile trials, the participants received the tactile stimulation at the same time intervals, while facing the other person, but no cube was presented. The whole task consisted of two blocks of 75 trials, each including 48 bimodal visuo-tactile, 12 unimodal tactile, and 15 catch trials, presented in random order to the participants. Each block lasted for about 5 min and the two blocks were intermingled with a 5-min break.

## Data analysis plan

In a first step, participants’ (baseline-corrected) reaction times (RTs) were submitted to a series of repeated-measure ANOVAs with Distance (D1–D6) as the within-subject factor and Power (powerless vs. powerful) as a between-subjects factor. To perform these analyses the two indices of the personal sense of power (i.e., the perception of one’s own power over others’ opinion and power over others’ behavior) were transformed into categorical variables—using a median split criterion (see Supplementary Materials for descriptive) – and contrast-coded variables (Power: powerless vs. powerful) were created. When the assumption of sphericity was violated as assessed by Mauchly’s test of sphericity, the Greenhouse–Geisser correction was applied. Post hoc comparisons were performed to investigate significant main and interaction effects.

To better characterize the spatial boundary of PPS and the near–far differentiation, similarly to what has been done previously in literature, the RTs for the six distances were fitted with a sigmoidal (Canzoneri et al. 2012; Serino et al. 2015b) and a linear (de Haan et al. 2016; Noel et al. 2019) function at the individual-subjects level.

In the case of sigmoidal fitting, the RTs are fitted according to the following equation:

$${\text{RT}}\left( {\text{D}} \right) = \frac{{{\text{RT}}_{\min } + {\text{RT}}_{\max } .e^{{\left( {D - central po{\text{int}} } \right)/slope}} }}{{{1} + e^{{\left( {D - central po{\text{int}} } \right)/slope}} }}$$

where Rt_min_ and Rt_max,_ respectively, indicate the ceiling and floor value of the sigmoidal function. Rt_min_ and Rt_max_ are fixed at the individual level by setting them, respectively, to the minimum and maximum reaction time for that subject, so the two free parameters extracted by the fit are *Cp* and b. In particular, one value “Cp” is the “central point”, namely the point where the sigmoidal function is maximally steep and can be used as a proxy of where the transition between near and far space occurs, i.e., the location of the PPS boundary.

In the case of linear fitting, the RTs at the six distances D were fitted with the following function:

$${\text{RT}}\left( {\text{D}} \right)\, = \,{\text{intercept}}\, + \,{\text{slope}}*{\text{D}}$$

The slope *b* quantifies the effect of distance on tactile processing, providing a marker of the amount of differentiation between peri- and extra-personal space. Higher values indicate a stronger modulation of multisensory processing as a function of the position of the external stimulus in space, suggesting a stronger differentiation in the multisensory processing of the near (around the body) and the far (and close to the other person) space.

## Results

### Pre-processing of reaction times

In line with previous studies, using the same visuo-tactile interaction task (e.g.(Pellencin et al. 2018)) or a similar PPS task (e.g., Canzoneri et al. 2012; Serino et al. 2015a), we only relied on reaction times (RTs) to the tactile stimulations to calculate indices of PPS representation. RTs 2 standard deviations higher or lower than the average participant’s RT were treated as outlier responses and excluded from the analysis. In line with previous studies using similar PPS tasks (e.g., Canzoneri et al. 2012; Serino et al. 2015a), we removed less than 2% of trials on average in all conditions. To obtain a general measure of multisensory processing, while controlling for a possible expectation effect due to the temporal delay of tactile stimulation, for each participant multimodal baseline-corrected RTs were calculated by subtracting the fastest unimodal RT from the mean of the RTs obtained to multimodal trials at each distance of the visual stimulus. Consequently, negative baseline-corrected RTs indicated multisensory facilitation. The multisensory effect is represented by a speeding effect of RT in the multisensory condition as compared to the fastest unimodal response, thus adopting a most conservative criterion to identify a facilitation effect (see Supplementary Materials, section “1.3 Preliminary analyses: testing the validity of the PPS social task” for comparison between unimodal and visuo-tactile RTs as a task sanity check).

## Testing the effect of the personal sense of power on social PPS representation: results and discussion

**Fig. 2:**
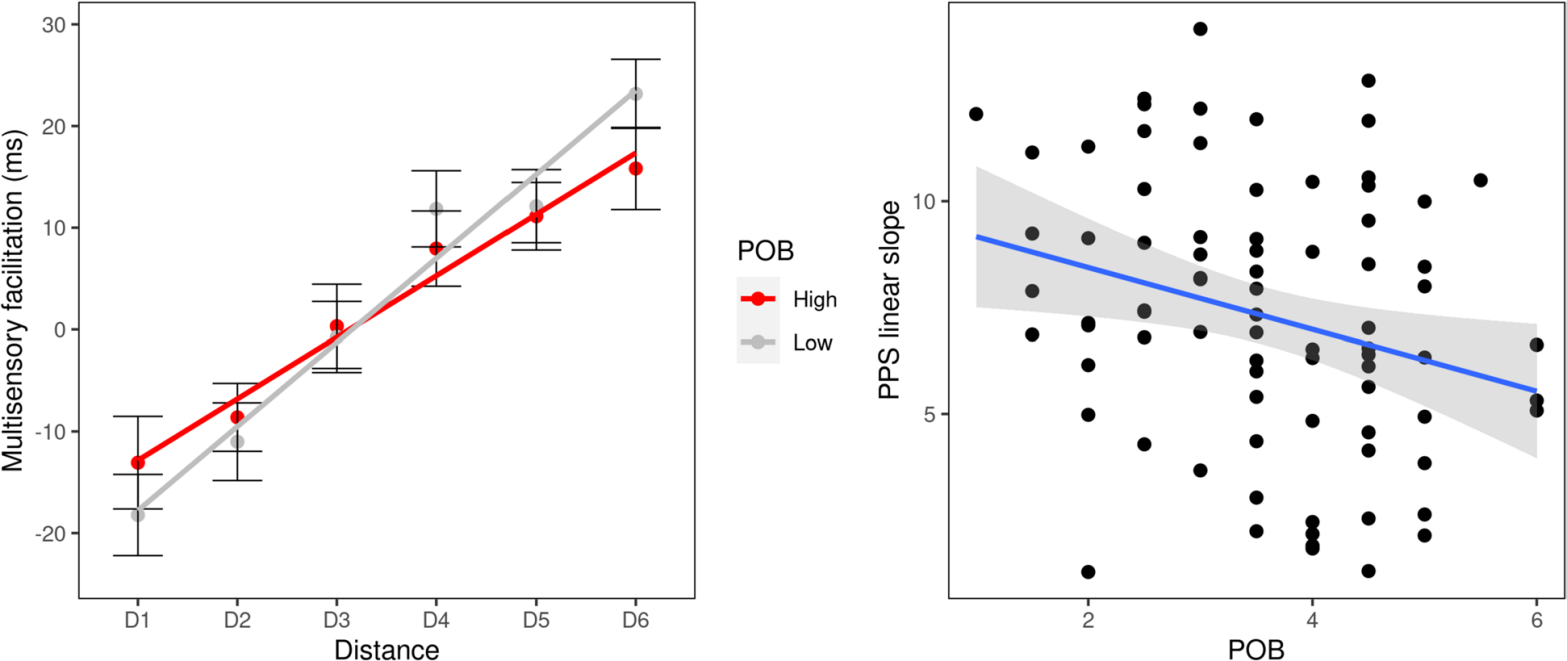
Study 1: PPS representation in a social context in function of Power on Others’ Behavior (POB). In the left plot, we show multisensory facilitation as a function of the distance of the visual stimulus, in the high and low POB groups (red and gray. respectively). Multisensory facilitation is obtained by averaging tactile unimodal and multisensory reaction times by distance and then subtracting the fastest unimodal response to multisensory reaction times. Error bars represent SEMs. To illustrate the interaction emerging from the two-way ANOVA (Power × Distance), we represent the linear fitting on multisensory facilitation in the two groups with solid lines. In the right plot, we illustrate the dependence of the linear slope of PPS on POB for individual subjects. Each dot represents a single participant’s PPS linear slope. To illustrate the significant effect of POB on the PPS slope, we overlay the linear fit (solid blue line) with its 95% confidence interval (gray shaded area)

The Distance × Power ANOVA testing the effect of the perception of one’s own power over others’ behavior yielded a significant main effect of Distance [*F*(5, 410) = 90.92, *p* < 0.001, *η*^2^ = 0.204]. This confirms that an approaching visual stimulus speeded up tactile processing when the former was within a given distance from the body (RTs at D1 and D2 were faster than RTs at D4, D5, D6, all *p *values < 0.001, which were not different from each other, all *p *values > 0.13). Importantly, this effect was qualified by the two-way interaction [*F*(5, 410) = 2.51, *p* = 0.029, *η*^2^ = 0.007] (Fig. 2, left panel). When performing distance-wise *t* tests comparing the powerful and powerless group, no statistically significant difference emerged (all *p *values > 0.10; Bonferroni corrected). This suggests that the difference between the participants who felt powerful (vs. powerless) on others’ behaviors evidenced by the ANOVA lies in global properties of the PPS response curve, rather than a difference at a single distance. Thus, the effect can be better investigated through the fitting parameters.

The parameters for the slope and the central point were extracted from the linear and the sigmoidal fit of the single-participant’s RTs curves, respectively. We used these parameters as dependent variables in two linear regressions in which the personal sense of power as the perception of one’s own and power over others’ behavior (POB) was entered as predictors:

$${\text{slope}}\,\sim \,{\text{A}}\, + \,{\text{B}}*{\text{POB}}{.}$$

Four out of the 84 participants were excluded from the analysis for having slope values higher/lower than two standard deviations from the mean.^2^ The perception of one’s own power over others’ behavior significantly predicted the individual slope of the curve of the linear fit [*R*^2^ = 0.0719, *F*(1,78) = 6.046, *p* = 0.016] (Fig. 2). The estimated coefficient (*B* = − 0.726, SE = 0.295, *β* = − 0.268 *p* = 0.016) was negative, indicating that the more the capacity to influence others behavior, the less steep the RTs curve becomes (Fig. 2, right panel). This suggests lower differentiation in the multisensory processing between the PPS and the far space—i.e., around the other person—as the feeling of having power over others’ behavior increases.

Given that powerful people are also higher in self-esteem, we ran an additional regression analysis including the self-esteem score as a covariate. The model was significant [*R*^2^ = 0.11, *F*(2,79) = 4.9, *p* = 0.01] and the coefficient for self-esteem was positive, although it did not reach the conventional value of statistical significance (*B* = 0.908, SE = 0.526, *β* = 0.186, *p* = 0.088). Nevertheless, the coefficient associated to power over others’ behavior was still significant (*B* = − 0.731, SE = 0.292, *β* = − 0.270, *p* = 0.014). Finally, no significant results emerged from the analyses on the central point of the sigmoidal fitting as the dependent variable. This excludes a more extended PPS representation for powerful individuals.

When we entered in the analysis the other index of personal sense of power that is the perception of one’s own power over others’ opinions, the ANOVA showed only the main effect of Distance [*F*(5, 410) = 94.16, *p* < 0.001, *η*^2^ = 0.216], but no interactions (*p* = 0.63). Regressions on the slope (both *p *values > 0.09) and the central points (both *p *values > 0.47) yielded similar non-statistically significant results.

To sum up, in Study 1 we investigated the role of the personal sense of power in the representation of PPS in a social context (i.e., when facing a person). We found that the perception of one’s own power over others’ behavior was linked to participants’ PPS representation.

When facing another person, results revealed a flatter PPS representation for powerful participants, while no extension in its size emerged. These findings suggest a reduced near–far differentiation for powerful participants, indicating an amount of multisensory processing allocated also in the far space, which corresponded to the space of the other person.

The perception of one’s own power over others’ opinion did not affect PPS representation. We will discuss this finding further in the general discussion. In the next study we tested whether power, if properly primed, affects PPS also in non-social context, i.e., when no person is present in the scene.

## Study 2

Study 2 was designed to further investigate under which circumstances the personal sense of power affects the PPS representation. The results of Study 1 show that the perception of one’s own power over others’ behavior modulated the representation of PPS when facing another person, i.e., in a social context. This opens the question of whether the personal sense of power has to be relevant in the context to shape PPS representation. The presence of another person in Study 1 could have made this personal disposition relevant and therefore cognitive accessible in the specific context. To further investigate the role of cognitive accessibility, in Study 2 we assessed participants’ PPS representation—by using the same visual–tactile interaction task as in Study 1—in a non-social context (i.e., an empty corridor) twice, before and after asking participants to remind an episode related to power. This reminder should make the participants’ beliefs about her/his personal sense of power (i.e., ability to influence and control others) cognitively accessible. If the influence of the personal sense of power over others’ behavior would emerge only in situations and contexts that make these individual beliefs relevant and cognitively accessible, we should find no difference in the PPS representation of powerful and powerless participants in the first assessment, whereas PPS should vary after the reminder as a function of the personal sense of power. Consistently with Study 1 results, we expected that only the perception of one’s own power over others’ behavior should reflect in a less differentiated PPS representation (in case its effect appears also in a non-social context) or only after the reminder (in the case, this individual disposition is relevant and cognitively accessible). This experimental design allows also to verify if powerful (vs. powerless) individuals have a general tendency to represent the near and the far space as less differentiated (independently of the specific context they are in); in this case, no modulation of the reminder should be found. Finally, note that asking to remind an episode related to power is used in social psychological studies to prime a temporary state of powerfulness (vs. powerlessness). Therefore, the effect of the reminder could add to the personal sense of power and intensify the participants’ feeling of being powerful or powerless (Chen et al. 2009). To check for this assumption, the personal sense of power in the two domains (on others’ behaviors and on others’ opinions) was also assessed before and after the reminder.

## Materials and methods

### Participants

Participants were 35 students (30 females; age ranging from 19 to 23 years, *M* = 20.29 and SD = 0.72) at the University of Trento. They were recruited among students who volunteered to participate in experiments in exchange of course credits. Informed consent was obtained from each participant.

## Materials

The PPS task used in Study 2 was identical to the task employed in Study 1 with the only difference being that the stimuli were presented in a pre-recorded scene of a virtual empty corridor (non-social context condition). The task was administered with the aid of a virtual reality headset (Oculus Rift DK2; 900 × 1080 per eye, ~  105° FOV) and the RealiSM software (Reality Substitution Machine, http://lnco.epfl.ch/realism).

## Procedure

The participants who volunteered received a link to an online questionnaire that included a series of scales among which was the Personal Sense of Power Scale (Anderson et al. 2012; Paladino et al. 2022). In a different e-mail, they were also invited to participate in a lab study on “The role of memory of emotional events in a cognitive task”.

**Fig. 3:**
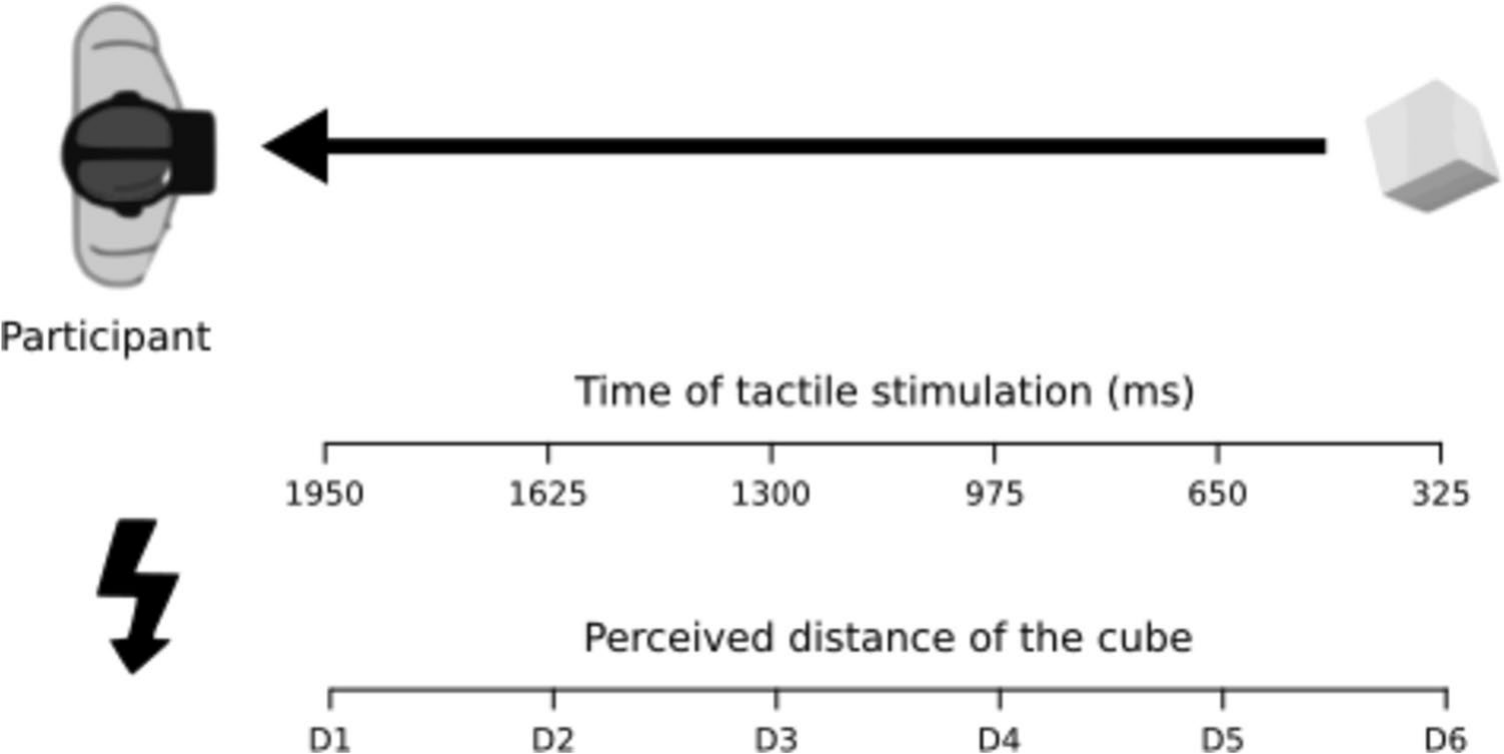
Study 2, experimental design. The experimental setup is identical to the one used in Study 1, with the exception that the actress was removed, and participants only saw the empty white corridor in front of them. In Study 2, participants performed the PPS task twice, before and after being asked to remind an episode in which they felt powerful vs. powerless

The laboratory session took place at least one week later. When in the laboratory, participants responded to a non-social PPS task twice, before and after they were asked to remind a personal episode. The PPS task was identical to the Study 1 task with two exceptions: no person appeared at the end of the corridor shown in the VR and the tactile stimulation was delivered on the trunk, at the level of the sternum (Fig. 3). Once completed the PPS task, half of the participants (randomly assigned) were asked to recall and write down an episode in which they felt powerful, the other half had to describe an episode in which they felt powerless.

 This was followed by some questions concerning the experience in VR and the scale of Personal Sense of Power (see Supplementary Materials, 2.1 Personal sense of power).

Note that only 27 (out of 35) participants completed the Personal Sense of Power scale before the laboratory session. To verify whether reminding an episode in which the participant felt powerful (vs. powerless) affected the idiosyncratic personal sense of power, each index was subjected to a Time (before vs. after the laboratory session) × Remind (powerful vs. powerless episode) ANOVA. No main effects or interactions emerged for any of the indices of power (all *p *values > 0.18). These results indicate that, differently from Chen and colleagues (Chen et al. 2009), reminding an episode of power did not influence participants’ sense of personal power on others’ opinion and others’ behavior, but likely made these abilities cognitively accessible.

## Results

### Pre-processing of reaction times and data analyses plan

Consistently with Study 1, RT outlier responses were removed, and baseline-corrected responses for the six distances were calculated (see Supplementary Materials for comparison between unimodal and visuo-tactile RT as a task sanity check, 2.2 Preliminary analyses: testing the validity of the PPS task). As far as the personal sense of power is concerned, as in Study 1, the two indices (average)—the perception of power over others’ behavior and over others’ opinion—were calculated from participants’ responses obtained in the online questionnaire and at the end of the experiment.

Then, baseline-corrected RTs were submitted to repeated-measure ANOVAs with Distance (D1–D6) and Session (before vs. after the reminder) as the within-subject factors, and participants’ Power (powerful vs. powerless over others’ behavior or over others’ opinion, in the first and second set of ANOVAs, respectively) and Reminder (Powerful vs. Powerless reminder) as the between-subject factors. Post-hoc comparisons were performed to investigate possible main and significant interaction effects.

Consistently with Study 1, then the RTs were fitted and the slope for the linear function and the central point for the sigmoidal function were extracted as parameters and used as dependent variables in the regression analyses. Similarly, to Study 1, for each index of the personal sense of power (calculated on the responses at the end of the experiment so as to include all participants), a contrast-coded variable was created, on the basis of the median split (see SM for descriptive), and entered as a factor in the following ANOVAs.^3^

To verify the contribution of other potential factors modulating PPS representation, additional analyses were carried out including as covariates participants’ age, height, and weight (see Supplementary Materials, 2.3 Additional analyses: age, height, and weight).

## Testing the effect of personal sense of power on non-social PPS representation

First, results from the ANOVA indicated that responses speeded as the stimuli approached [main effect of Distance: *F*(5, 155) = 23.85, *p* < 0.001, with Greenhouse–Geisser correction, *η*^2^ = 0.087], thus showing the predicted facilitation effect.

More interestingly, a significant three-way interaction Power (over others’ behavior) × Session × Distance emerged, [*F*(5, 155) = 3.35, *p* = 0.007, *η*^2^ = 0.008], thus highlighting that this facilitation effect varied in function of the session and the reminder of the powerful vs. powerless episode. No other significant effects emerged from this analysis. Namely, since the type of reminder did not show any significant effect (4-way interaction, *p* = 0.37, all other *p *values > 0.36), we excluded this factor from further analyses. Indeed, a three-way (Power × Session × Distance) ANOVA still showed a significant Power × Session × Distance interaction [(*F*(5, 165) = 3.75, *p* = 0.003, *η*^2^ = 0.009].

To better understand the interaction, baseline-corrected RTs to the first and the second PPS task were entered separately in two Power × Distance ANOVAs. In the first PPS session, Power over others’ behavior did not play a role, as only a main effect of Distance emerged [*F*(5, 165) = 21.31, *p* < 0.001, with Greenhouse–Geisser correction, *η*^2^ = 0.117].

**Fig. 4:**
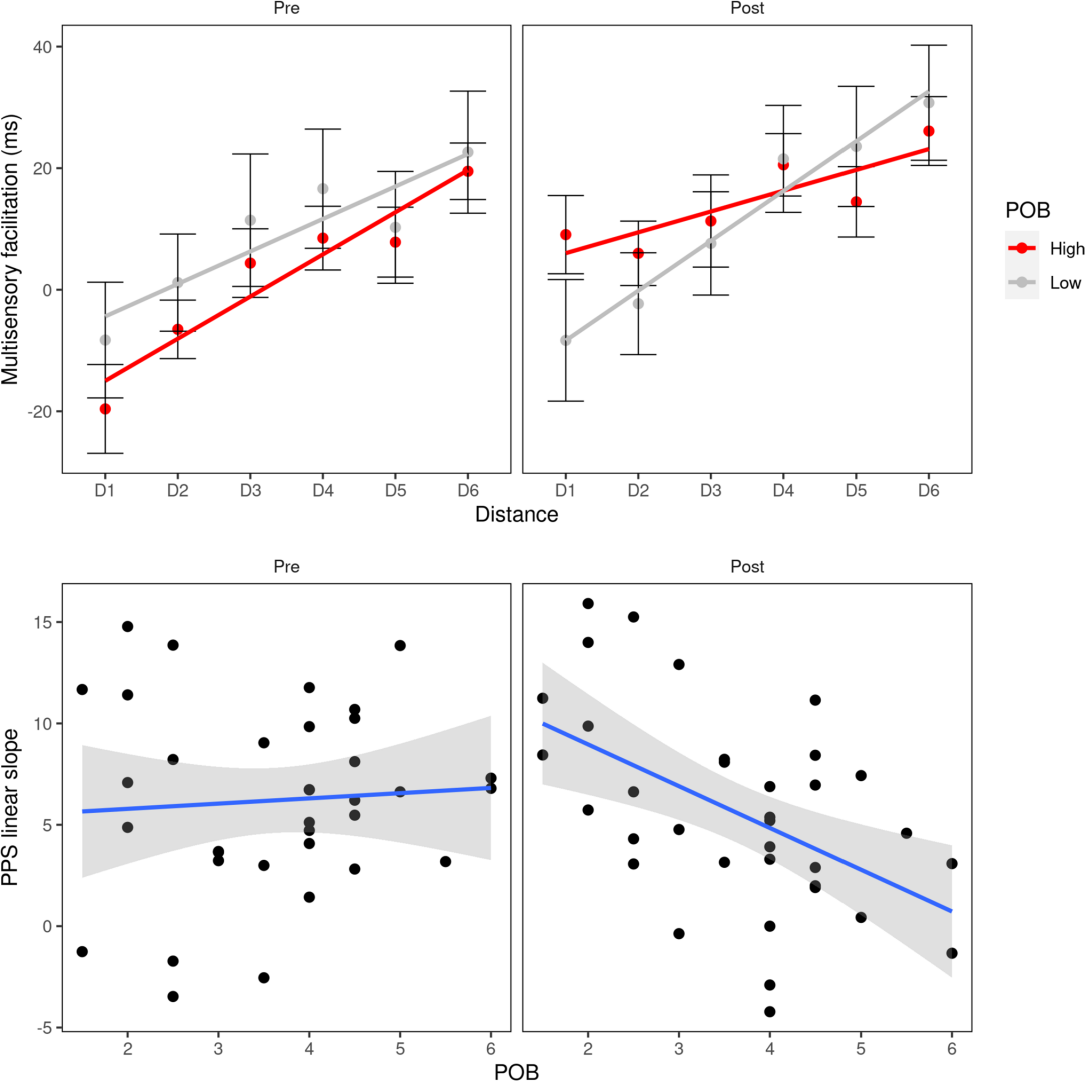
Study 2, PPS representation in a non-social context in function of power over others’ behavior (POB) and before and after the reminder. In the top plots, we show multisensory facilitation as a function of the distance of the visual stimulus, in the high and low POB groups (red and gray, respectively), and before and after the reminder (left and right plots, respectively). Error bars represent SEMs. We represent the linear fitting on multisensory facilitation in the two groups with solid lines. In the bottom plots, we illustrate the dependence of the linear slope of PPS on POB for individual subjects, in the pre- and post-reminder sessions (left and right plots, respectively). Each dot represents a single participant’s PPS linear slope. Solid blue lines represent linear fits with its 95% confidence intervals (gray shaded area). Both when analyzing PPS representation at the group level (top plots) and at the individual level through linear fitting (bottom plots), significant modulations of PPS representations were observed only in the post-reminder session

In the second PPS assessment (after participants were asked to remind an episode related to Power), the effect of Distance, [*F*(5, 165) = 18.49, *p* < 0.001, *η*^2^ = 0.1], was qualified by the significant Power × Distance interaction, [*F*(5, 165) = 3.31, *p* = 0.012, *η*^2^ = 0.02] (Fig. 4). Similarly, to results from Study 1, distance-wise *t* tests between individuals in the powerful and powerless groups yielded no significant results (all *p *values > 0.10). As in Study 1, we focused thus on the fitting analysis for the interpretation of the effects. As the ANOVA revealed a three-way interaction, we relied on the following linear mixed model:

$${\text{Slope}}\,\sim \,{\text{A}}\, + \,{\text{B}}*{\text{POB}}\, + \,{\text{C}}*{\text{Session}}\, + \,{\text{D}}*{\text{Session}}*{\text{POB}}{.}$$

Worthy of note, the equation includes as predictors the individual score on Power over others’ behavior (POB), the Session (before vs. after the reminder) of the task where the PPS parameters were extracted, and the interaction between these two terms. To assess the significance of the mixed models, we performed likelihood ratio tests against a null model including just the individual intercepts for each participant. The model was significant [*χ*2(3) = 13.875, *p* = 0.003] and the interaction between Session and POB was significant (*D* = 2.3, SE = 0.681, *p* = 0.0017 using Satterthwaite approximation to degrees of freedom), meaning that the relation between POB and PPS slope was modulated by the reminder. To characterize the interaction, we performed two linear regressions for the pre- and post-reminder parameters of the RTs curve separately, by fitting the same model as for Study 1:

$${\text{Slope}}\,\sim \,{\text{A}}\, + \,{\text{B}}*{\text{POB}}{.}$$

Before the reminder, POB was not linked to the PPS slope (*p* = 0.698). After the reminder, the model was significant [*R*^2^ = 0.261, *F*(1,33) = 11.64, *p* = 0.0017] and the coefficient estimate was negative (*B* = − 2.06, SE = 0.603, *β* = − 0.51, *p* = 0.0017): higher POB values corresponded to a flatter slope of the curve representing individuals’ PPS representation (Fig. 4). No significant effects were found when the same analysis was run on the central point of the sigmoidal fitting.

When we analyzed the other facet of the personal sense of power that is the perception of one’s own power over others’ opinion (POO), the ANOVA yielded only a main effect of Distance [*F*(5, 165) = 30.67, *p* < 0.001, *η*^2^ = 0.11] with Greenhouse–Geisser correction, and a marginally significant the three-way interaction Power × Session × Distance, [*F*(5, 165) = 2.36, *p* = 0.061, with Greenhouse–Geisser correction, *η*^2^ = 0.006]. (For more details on this analysis, see SM). A linear mixed model including POO and the interaction term as predictors was only marginally significant [*χ*2(3) = 6.93, *p* = 0.074].

To sum up, in Study 2 we investigated the role of the personal sense of power over others’ behavior and of power others’ opinion on PPS representation in a non-social context. Across the ANOVA and the regression, and similarly to Study 1, we found that the perception of one’s own power over others’ behavior is associated with a less sharp differentiation, in terms of multisensory processing, of PPS and extra-personal space representation in a non-social context, but only after participants were asked to remind an episode of power. This result suggests that personal sense of power over others’ behavior does not affect PPS representation tout-court, but only whenever cognitively accessible or contextually relevant.

## General discussion

Does feeling powerful affect self-representation in space? To address this question, in the present research we took advantage of the concept of PPS to investigate whether (Study 1) and when (Study 2) the personal sense of power affects the individual multisensory representation of space. In both studies, we relied on a visual–tactile interaction task to gauge PPS representation and on a self-report scale to assess participants’ personal sense of power in two different domains: others’ behaviors and others’ opinions. We focused our analysis on investigating the modulation of power on the PPS representation both in terms of its spatial extent (i.e., *where* the transition between the representation of the peri-personal and the extra-personal space occurs, the PPS boundary) and its shape (i.e., how differentiated peri- and extra-personal space are, resulting from the amount of multisensory processing allocated in the near vs. far space).

In Study 1, where another person was in the far space simulating the context of social interaction, we found that feeling powerful over others’ behavior was negatively associated with the steepness of PPS representation, suggesting that PPS was less differentiated (from the far space occupied by the other) as power increased. In Study 2, we further investigated the role of the personal sense of power by assessing participants’ PPS representation in a non-social context (i.e., an empty corridor, with no other person being present). We found that recalling an episode related to power—aimed at making the personal sense of power temporarily cognitively accessible—strengthened the negative correlation between feeling powerful over others’ behavior and the PPS slope. This suggests that, even when no other person is present during the assessment, a reduced near–far differentiation emerged—with an increased amount of multisensory processing allocated to the far space—when the participant’s sense of power is made accessible*.*

Taken together, the present findings suggest a positive answer to our initial question. Feeling powerful over others’ behavior modulates multisensory self-representation in space. The area surrounding the body and mapped as part of the self is not systematically more extended, as we found no effect on the central point, the index normally considered a hallmark of PPS extent. However, this area is represented, in terms of multisensory processing, as less differentiated from that of the far space in powerful individuals. This conclusion is supported by a significant relationship between the slope of the PPS function and sense of power. Difference between slopes has been sometimes interpreted as differences in PPS size (see e.g., de Haan et al. 2016); we suggest that—in the absence of a change in the central point—such difference is a proxy of the definition of PPS, being sharper at steeper slopes and shallower at flatter slopes (see e.g., Serino et al. 2021; Ellena et al. 2022). In this context, the present results can be interpreted as the perception of power on others’ behavior modulates the distribution of multisensory processing in space. This is more confined in the near space for powerless individuals and more distributed across near and further positions of space as the feeling of power over others’ behavior increases. This finding could be the result of the minor importance of spatial distance per se or the outcome of a more dynamic representation of space in powerful individuals: they would extend and contract PPS representation—moment by moment—so as to dynamically monitor not only their own space, but also that occupied by the others they perceive they can influence. Thus, rather than something intrinsic to the individual, this lower differentiation between close and far space appears to be a downstream consequence of feeling of having power over others’ behaviors.

Why does feeling power over others’ behavior alter self-representation in space? One of the functions of the PPS representation is to support action. Less differentiation between PPS and extra-personal space could be interpreted as a consequence of a more distributed mapping of space to potentially prime actions with respect to external objects within a larger space, i.e., an enhanced power for action that typically characterizes power as a psychological state. Approach motivation can be defined as the energization of behavior oriented toward the desired goal. In this specific case, the goal would be consolidating power by influencing others’ behavior. This reduced PPS differentiation would then indicate that the person is preparing to act in the space of social interaction, which is the area that separates one’s own from the body of the potential target of influence. Although theoretically sound, this consideration remains only speculative, as the present study does not provide empirical evidence supporting it.

An important contribution of the present study is to investigate two dimensions of personal sense of power. Power is a complex concept that has been defined and operationalized in several ways. In the present research, we focused on the perception of power over others’ opinions and over others’ behavior and found that only the latter was consistently related with PPS representation. These two indices differ in several respects, and this might have contributed to this differential effect. At the empirical level, both in Study 1 and in Study 2, the index of power over others’ behavior was normally distributed (whereas the power over others’ opinion index was right-skewed), with the median value at the midpoint of the scale (neither agree nor disagree). The index of power over others’ behavior likely best captured a significant variation among participants’ personal sense of power (i.e., participants above the midpoint agreed with statements expressing power over others, those below disagreed with such statements); this could have contributed to the present results. In addition to these empirical considerations, also conceptual differences could underlie the effect we found. Previous research has shown that, although both indices are related to a dominant personality, only the perception of having power over others’ behavior is linked with the motivation to lead (e.g., giving orders and get things going; enjoying having authority over people”, Paladino et al. 2022). Power over others’ behavior, compared to opinions, involves thus the dimension of control and agency over another person’s body. These differences could explain the selective effect of power over others’ behavior on PPS representation.

What are the implications of the present research for the literature on PPS? Our findings extend current knowledge on PPS representation at least in three respects: (1) they show that the shape of PPS representation maps also onto a personal sense of power over others’ behavior—a psychological characteristic different from those previously investigated in the context of PPS; (2) they provide some new insights on how and when individual differences contribute to PPS representation; (3) they offer further evidence on the role of PPS representation in social interactions.

Previous studies have shown that the extent of PPS representation is linked to traits like anxiety (Sambo and Iannetti 2013), claustrophobia (Lourenco et al. 2011; Hunley et al. 2017) or empathy (Gherri et al. 2022). The personal sense of power over others’ behavior differs from these characteristics in many respects; it is a subjective belief in one’s own relational ability, with no clinical implications and not connected to body conditions and symptoms. In this respect, our research does not only add a new variable to the list of factors influencing PPS representation, but it also shows that a belief—having a top-down influence on the way we approach and interpret the external world—can affect the coding of the space surrounding the body.

The present research also provides some new insights on the role of individual psychological differences in the PPS representation, suggesting that the influence of psychological traits—and eventually other personal beliefs—is context dependent. In our studies, we found that participants’ self-reported ability to influence others behavior predicted their PPS representation, when the context made the personal sense of power salient, (e.g., after a reminder or as another person is present in the space). These findings echo other studies showing modulation of PPS representation as depending on individual differences (phobias), only in relevant contexts (e.g., when specific fearful stimuli—as spiders (de Haan et al. 2016) or dogs barking (Taffou and Viaud-Delmon 2014) were present).

Overall, these studies suggest that the psychological individual difference must be relevant in the situation to account for some inter-individual variation in PPS representation. If this reasoning is correct, future studies investigating individuals’ variability in PPS representation need also to pay attention to “where” the individual is and “what/who” is present in this environment.

A related implication of the above-discussed context-dependent effect is that PPS representation is more likely to vary across contexts in some individuals than others. In Study 2, we found that the PPS representation of participants’ feeling powerful over others’ behavior was more malleable compared to that of powerless participants, as in the first and not in the latter group, PPS varied after the reminder of power. To the best of our knowledge, between-individuals and within-context variations in PPS representation have been investigated at the same time only in a study (Hunley et al. 2017). Relying on line-bisection task, Hunley et al. found that participants high (vs. low) in claustrophobia had a more extended PPS representation and showed less expansion when using a tool that allowed reaching further locations of space. Similarly, to the present research, such result suggests that individual differences not only predict the shape of PPS, but also the flexibility of such representation across contexts or the type of interaction with the environment. In this respect, the present findings add a further element to our understanding of PPS as sensory–motor interface mediating individual–environment interactions. Not only sensory inputs, action possibilities, or objective characteristics, but also individual perception and interpretation of the environment affect PPS representation and explain its plasticity across contexts.

Finally, our findings provide further evidence on the social modulation of PPS representation and its role in an interpersonal context. Previous research has showed that positive characteristics of others, such as morality (Iachini et al. 2015a; Pellencin et al. 2018), cooperation (Heed et al. 2010; Hobeika et al. 2019) or fairness (Teneggi et al. 2013) lead to an extension of individuals’ PPS representation. The present research adds to these findings by showing that power over others’ behavior, an important feature structuring our social interactions, goes hand in hand with the tendency to differentiate less, in terms of multisensory processing, what occurs close vs. far to one’s own body. Conceptually, power is not comparable to any of the social features previously investigated. However, power, along with cooperation, and positive perception increase the chances to approach and interact with the other person involved in the interaction. In this regard, our research supports the idea of the role of PPS to support action (Ladavas and Serino 2008; Bufacchi and Iannetti 2018) or, more specifically, the interaction between the individual and the environment (Serino 2019).

To conclude, power affects self-representation in space. People feeling powerful over others’ behavior differentiate less between the space surrounding the body and the far space, when in presence of another person or in contexts in which personal sense of power is made cognitively accessible.

### Supplementary Information

Below is the link to the electronic supplementary material.

Supplementary file1 (DOCX 108 KB)

## Data Availability

The data that support the findings of this study are available from TB upon reasonable request.

## Footnotes

1 Paladino et al. (2022) investigated the factorial structure of the Personal Sense of Power scale and its validity in two studies. In Study 1, the analyses were conducted on the responses of 148 individuals, 119 of whom participated in the studies included in the present research. Note this factorial structure was replicated in Study 2 (*N* = 231).

2 As a control, we ran an alternative analysis to identify potential outliers, excluding subjects having a Cook’s distance larger than 4/*n* on the POB regression. The analysis excluded again four subjects. Three of these four were also excluded by the analysis based on standard deviation. The regression on POB was still significant [*R*^2^= 0.1, *F*(1,78) = 8.6, *p* = .004].

3 Half of the participants (randomly assigned) were asked to recall and write down an episode in which they felt powerful and the other half had to describe an episode in which they felt powerless, and after that they were further divided on the basis of the median split resulting in this distribution: 6 of the 19 participants in the high POB group experienced non-powerful manipulation, while 5 of the 16 participants in the low POB group experienced powerful manipulation.
