# Supplementary material for: Space for power: feeling powerful over others’ behavior affects peri-personal space representation
Source: Exp Brain Res. 2023 Oct 21;241(11-12):2779–93. doi: 10.1007/s00221-023-06719-1 (PMC10635978; doi:10.1007/s00221-023-06719-1)
Supplement: Supplementary file 1 — Supplementary file1 (DOCX 108 KB) [file 221_2023_6719_MOESM1_ESM.docx]

**Supplementary materials**

1. **Study 1**

**1.1 Recruitment and data collection**

In Study 1 we included in the analysis 84 participants who took part in studies on peripersonal space in our lab in the period November 2016-April 2017. Participants were recruited among those who volunteered to studies in social psychology in exchange for course credits. Students who volunteered received also a link to an online questionnaire; this included the personal sense of power scale and the core self-evaluations scale – of interest for the present study-, but also other scales that were of interest to other researchers in the department. A few days later, they could receive an invitation to participate in a study on PPS (others were invited for other studies). Independently of the specific goal, each study started with a social PPS task (full description in the manuscript). Worthy to note here, no information on the person they saw in the virtual reality was provided. The responses to this social PPS task and the questionnaire in which an impression of the person was provided were included in the analysis. Manipulation, and other measures, tasks could follow. The data were collected by 4 experimenters; all were instructed by the same person (the second author of the manuscript) and followed the same instructions to present the PPS social task. The data were collected in a single institution; the same lab, virtual reality headset (Oculus Rift DK2; 900x1080 per eye, ~105° FOV), and software to present the stimuli (RealiSM software, Reality Substitution Machine, <http://lnco.epfl.ch/realism>) and register the reaction times were used.

**1.2 Personal sense of power: Power over Others’ Behavior and Power over Others’ Opinion.**

Based on Paladino, Stefani, and Perinelli (2022), scores for Power over Others’ Behavior (POB) and Power over Others’ Opinion (POO) were calculated for each participant (see Table S1).

Table S1

*Power over Others’ Behavior (POB) and Power over Others’ Opinion (POO): Descriptive Statistics and Correlations in Study 1*

| Subscale | Mean | Median | SD | Median Absolute Deviation | POO | POB |
| --- | --- | --- | --- | --- | --- | --- |
| POO | 4.82 | 5.00 | 1.13 | 1.12 | _ | - |
| POB | 3.55 | 3.50 | 1.16 | 1.48 | 0.25 * | - |

*Notes.* N = 84; POO = Power over Others’ Opinion; POB = Power over Others’ Behavior * *p* < .05.

**1.3 Preliminary analyses: testing the validity of the PPS social task.**

In order to confirm that visuo-tactile trials showed a space-dependent multisensory facilitation effect with respect to unimodal tactile trails, the mean RTs to unimodal and bi-modal trials for each time interval of the tactile stimulation (hereafter “distance”) were calculated and entered in a repeated measure ANOVA with Modality (uni-modal tactile vs. bimodal visual-tactile trials) and Distance of the virtual object (D1-D6; D1 and D6 are the closest and the furthest distance from the participant) as within-subject factors. The main effect of Modality (*F*(1, 83) = 134.30, p < .001) and of Distance (*F*(5, 415) = 34.46, *p* < .001) were significant, and, as expected, qualified by a two-way Modality X Distance interaction (*F*(5, 415) = 8.39, *p* < .001). Simple effects (Bonferroni corrected) showed that multimodal trials were always faster than uni-modal trials (all *p’s* < .001) and followed a pattern indicative of a PPS: the processing of tactile stimulations was facilitated when the cube (the visual stimulus) was closer to the participant (RTs for all distances were significantly different from each other, all *p’s* < .001, except for D4 and D5). For unimodal trials, RTs did not follow a clear pattern; they were all equally fast (all *p*’s>0.1) except for D6. Responses to D6 were slower than all D’s, but not D4 (*p* = .09). These initial analyses replicated previous findings and showed the validity of the paradigm to assess PPS representation.

**1.4 Additional analyses**

In order to investigate the effect of power on detecting tactile stimuli, we regressed the mean RTs to unimodal and multimodal trials on POB. Results indicated that POB was not a significant predictor in both analyses (*p* = .27 for unimodal trials, *p* = .38 for multimodal trials).

As an additional control, we run the POO~slope regression on the slope obtained from unimodal RTs, which should only reflect spurious effects on RTs, unrelated to PPS representation. The regression was not significant at *p* = .2.

1. **Study 2**

**2.1 Personal sense of power: Power over Others’ Behavior and Power over Others’ Opinion.**

Scores for Power over Others’ Behavior (POB) and Power over Others’Opinion (POO) were calculated for each participant (see Table S2).

Table S2

*Power over Others’ Behavior (POB) and Power over Others’ Opinion (POO): Descriptive Statistics and Correlations in Study 2 (measured at the end of the task)*

| Subscale | Mean | Median | SD | Median Absolute Deviation | POO | POB |
| --- | --- | --- | --- | --- | --- | --- |
| POO | 5.33 | 5.60 | 0.88 | 0.89 | - | - |
| POB | 3.63 | 4.00 | 1.21 | 1.48 | 0.42 * | - |

*Notes.* N = 35; POO = Power over Others’ Opinion; POB = Power over Others’ Behavior * *p* < .05.

**2****.2 Preliminary analyses: testing the validity of the PPS task.**

To verify the validity of the task in measuring the extension of the PPS representation, and the eventual effect of repetition we submitted the mean RTs to unimodal and bi-modal trials for each time interval of the tactile stimulation (hereafter “distance”) in an Order of the task (first vs. second) X Modality (uni-modal tactile vs. bimodal visuo-tactile trials) X Distance of the virtual object (D1, D2, D3, D4, D5, D6) ANOVA, with all factors manipulated within-participants. The analysis yielded significant main effect for Modality, (F(1, 34) = 56.42, p < .001), Distance, (F(5, 170) = 13.87, *p* < .001), and Order (F(1, 34) = 18.96, *p* < .001); and significant interaction for Modality X Order, (F(1, 34) = 4.53, *p* = .04), and Modality X Distance, (F(5, 170) = 5.46, *p* < .001). These effects were qualified by a marginal significant three-way interaction, (F(5, 170) = 2.18, *p = .058)*. An Order X Distance ANOVA was conducted separately for RTs to Multimodal and Unimodal trials. For multimodal trials, we found that participants had faster RTs in the second (M = 336 ms , SD = 65 ms) than in the first task (M = 365ms, SD = 77.6 ms), as showed by the significant effect of Order (F(1, 34) = 18.95, *p* < .001). Importantly, the analyses yielded also a significant main effect of Distance (F(5, 170) = 31.01, *p* < .001). For unimodal trials, the significant main effect for Order showed that practice speeded up participants’ responses (M = 396 ms, SD = 96.2 ms and M = 359 ms, SD = 75.9 ms, when first and second task respectively), (F(1, 34) = 19.86, *p* < .001). The significant main effect of Distance (F(1, 34) = 19.86, *p* < .001) was due to slower responses to D6 compared to D5, *p* = .05, and D2, p = .09. All other Ds were not statistically different from each other, all *p*’s >.7.

Taken together these data showed that the paradigm was valid to assess PPS extension, but that repetition and practice speeded up participants’ responses both for unimodal and multimodal trials (although note that the effect was stronger for unimodal than multimodal trials).

**2.3 Post-hoc analyses**


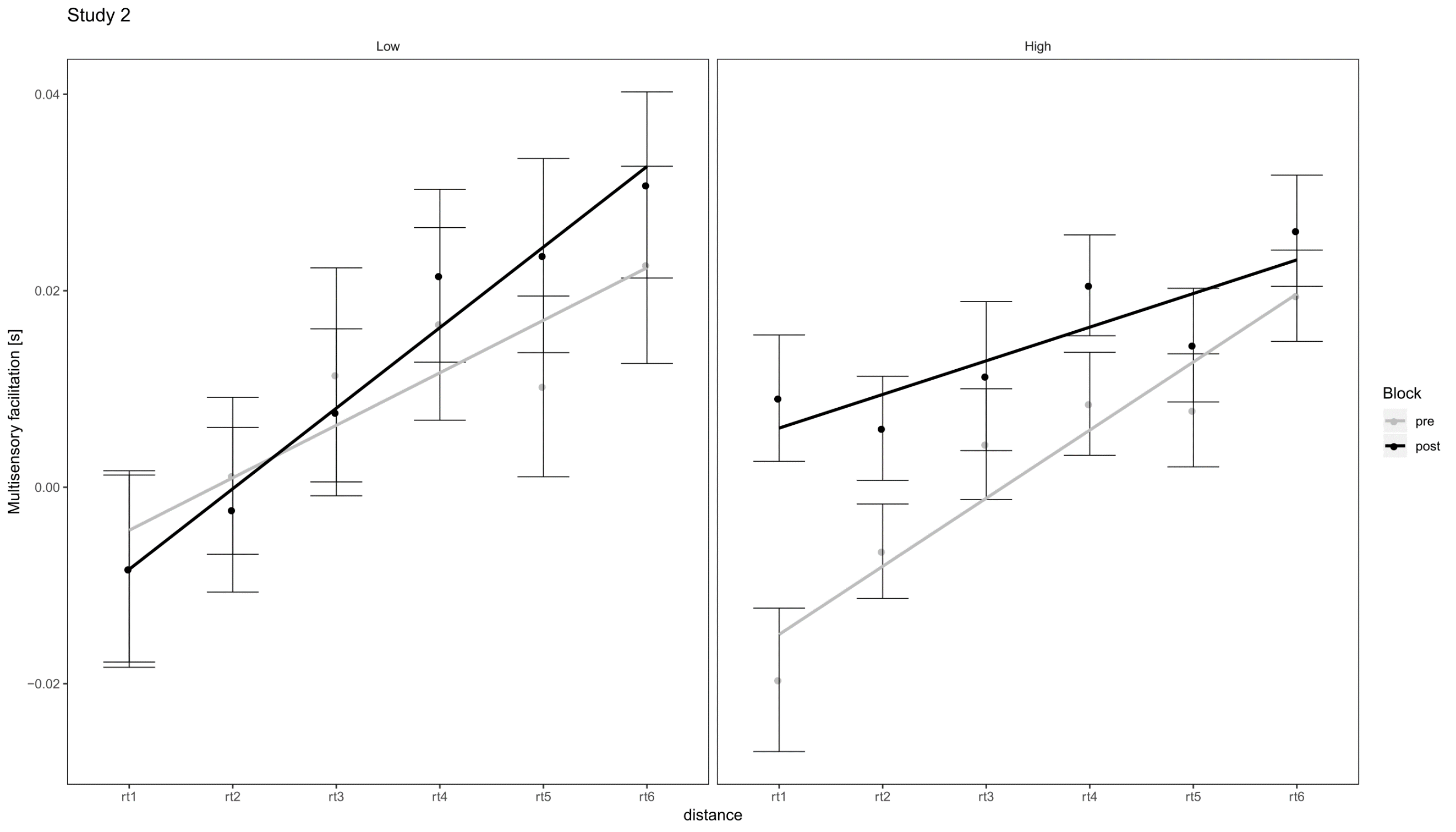

**Figure S1.** Study 2: effect of the experimental manipulation on low (left) and high (right) POB participants. Errorbars represent SEMs. To illustrate the interaction emerging from the two separate two way ANOVAs (Power X Distance), we represent the linear fitting on multisensory facilitation in the two groups with solid lines.

Here we present an alternative formulation of post-hoc analyses for the three-way ANOVA performed in Study 2, mentioned in the Discussion of the main text. If performing Order X Distance ANOVAs for powerful and powerless (in terms of the influence of others’ behavior) participants separately, we found that the reminder affected the PPS in high POB participants (F(5,90) = 3.69, *p* = .004), but only marginally significant for low POB participants (F(5,75) = 2.02, *p* = .09). This suggests that the PPS representation in more dominant individuals is more susceptible to the experimental manipulation.

We then investigated in more detail the marginally significant three-way ANOVAs reported in paragraph 3.2.2 of the main text. The regression on POO yielded a marginally significant three-way interaction (*p*=.061 with Greenhouse-Geisser correction). To investigate such an effect, similar to what was done for POB, we performed two separate Distance*Power ANOVAs on the pre- and post-reminder experimental sessions. In the pre-reminder session, the interaction Power*Distance was clearly not significant (*p* = .69). The effect seems to be driven mainly by an only marginally significant Power*Distance interaction in the post-reminder session (F(5,165) = 1.98, *p* = .101 with Greenhouse-Geisser correction). In distance-wise t-tests, we found that RTs were significantly different between high and low-power participants only in the post-manipulation session and only for D1, with RTs being significantly slower for high POO participants (*p*=.01, Bonferroni corrected).

**2.3 Additional** **analyses: age, height, and weight**

We compared age, height, and weight between the high and low POB and POO groups to analyze potential confounders. We ran additional analyses, comparing age, height, weight, and gender between the high and low POB and POO groups employing two-sample t-tests, except for gender in which we relied on a Chi-Squared test. We found no significant effect (all *p*-values >.1), except for a weakly significant effect of the POB group on age (participants were slightly older in the high vs. low POB group, *p* = .045) and a trend of POO on gender (more males in the high POO group, *p* = .1).

Therefore, we ran additional analyses, including these two factors – gender and age - as covariates in the POB~slope linear regression.

slope~ A+B*POB

The model including age as a covariate was significant (R2 = .076, *p* = 0.048), and so was the coefficient for POB (*p* = 0.014), while the coefficient for age was not significant (*p* = .57). Similar results were found including gender as a covariate. The model was significant (R2 = .079, *p* = 0.041) as the coefficient for POB (p = 0.013), while the coefficient for gender was not significant (*p* = .42).

Globally, these results indicated that the high- and low-power groups did not differ in height or weight, suggesting that the observed PPS modulation is not linked to one of these factors. In addition, it is worth mentioning that previous research – conducted on relatively large samples - found that correlations between height and sense of power were small in size (e.g. in Körner et al., 2021, the correlation between body height and sense of power ranged from *r* = .08, for men, to *r* = .01 for women).

**References**

Körner, R., Petersen, L. E., & Schütz, A. (2021). Do expansive or contractive body postures affect feelings of self-worth? High power poses impact state self-esteem. Current Psychology, 40, 4112-4124.

Paladino MP, Stefani S, Perinelli E (2022). Feeling of power: Validation of the Italian

Personal Sense of Power Scale. Psicol Soc 17:103–123
